# The Prognostic Value of Exercise Stress Echocardiography in Asymptomatic Moderate and Severe Aortic Stenosis: A Systematic Review of Stress-Derived Hemodynamic and Functional Markers

**DOI:** 10.3390/jcm15093247

**Published:** 2026-04-24

**Authors:** Andrea Sonaglioni, Michele Lombardo, Giulio Francesco Gramaglia, Gian Luigi Nicolosi, Massimo Baravelli

**Affiliations:** 1 Division of Cardiology, Istituto di Ricovero e Cura a Carattere Scientifico, MultiMedica, 20123 Milan, Italy; michele.lombardo@multimedica.it (M.L.); massimo.baravelli@multimedica.it (M.B.); 2 Department of Emergency, Fondazione Istituto di Ricovero e Cura a Carattere Scientifico, Ca’ Granda, Ospedale Maggiore Policlinico, 20122 Milan, Italy; giulio.gramaglia@unimi.it; 3 Division of Cardiology, Policlinico San Giorgio, 33170 Pordenone, Italy; gianluigi.nicolosi@gmail.com

**Keywords:** aortic stenosis, exercise stress echocardiography, risk stratification, asymptomatic patients, prognosis

## Abstract

**Background:** Risk stratification of patients with asymptomatic aortic stenosis (AS) remains challenging, as symptom-based assessment may underestimate disease severity. Exercise stress echocardiography (ESE) provides a comprehensive evaluation of valvular, ventricular, and cardiopulmonary responses under physiological stress and may improve prognostic assessment. **Methods:** A systematic review was conducted according to PRISMA guidelines to evaluate the prognostic value of ESE in asymptomatic moderate and severe AS. Electronic databases (PubMed, Scopus, and EMBASE) were searched from inception to March 2026. Studies were included if they assessed adult patients with asymptomatic moderate or severe AS undergoing exercise-based stress echocardiography and reported clinical outcomes. Studies using exclusively pharmacological stress or lacking outcome data were excluded. Data were extracted and synthesized qualitatively. Continuous variables were summarized as weighted medians and interquartile ranges. **Results:** A total of 11 studies were included, encompassing a heterogeneous population of patients with moderate-to-severe and severe AS. During follow-up, a substantial proportion of patients experienced adverse events, including symptom onset, aortic valve replacement, or death. Across studies, exercise-derived parameters consistently showed strong prognostic value. In particular, exercise-induced increases in mean transvalvular gradient, an elevated E/e’ ratio, the development of pulmonary hypertension, and reduced functional capacity emerged as the most reproducible predictors of adverse outcomes. Notably, thresholds such as an increase in mean transvalvular gradient ≥ 18–20 mmHg, peak exercise E/e’ ≥ 15, and systolic pulmonary artery pressure ≥ 60 mmHg were consistently associated with a higher risk across multiple studies. Myocardial deformation parameters and biomarkers such as exercise-induced BNP further contributed to risk stratification in selected studies. In contrast, resting parameters alone were less consistently predictive. **Conclusions:** ESE provides incremental prognostic information in asymptomatic moderate and severe AS by unmasking subclinical hemodynamic and myocardial abnormalities. The integration of stress-derived parameters, including reproducible threshold values, into clinical assessment may improve risk stratification and support more individualized management strategies. Further studies are needed to validate these cut-offs and define their role in guiding clinical decision-making.

## 1. Introduction

Aortic stenosis (AS) represents the most prevalent valvular heart disease in developed countries and its incidence is steadily increasing in parallel with population aging [1]. While the clinical management of symptomatic severe AS is well established, with aortic valve replacement (AVR) providing clear survival benefit, the optimal management of asymptomatic patients remains a matter of ongoing debate [2].

The natural history of asymptomatic AS is highly variable and often unpredictable. Although the annual risk of sudden cardiac death in truly asymptomatic individuals is relatively low, a substantial proportion of patients may experience rapid disease progression, symptom onset, or irreversible myocardial damage before clinical recognition [3]. Importantly, symptom assessment in this population is inherently challenging. Many patients, particularly older adults, may unknowingly limit their physical activity, leading to underreporting of symptoms and delayed referral for intervention [4]. As a result, reliance on symptom status alone may be insufficient to guide optimal timing of AVR [5].

In this context, there has been increasing interest in identifying objective markers capable of detecting early functional impairment and improving risk stratification in asymptomatic AS. Exercise testing is currently recommended by international guidelines to unmask latent symptoms and abnormal blood pressure responses [6,7]. However, conventional exercise testing provides limited insight into the underlying hemodynamic and myocardial mechanisms [8].

Exercise stress echocardiography (ESE) offers a more comprehensive approach by integrating functional, hemodynamic, and structural assessment during physiological stress [9]. This technique enables real-time evaluation of transvalvular gradients, left ventricular systolic and diastolic function, pulmonary pressures, myocardial deformation, and cardiopulmonary interaction [10]. Through this multiparametric assessment, ESE has the potential to uncover subclinical abnormalities not detectable at rest, thereby providing incremental prognostic information [11].

Over the past two decades, several studies have investigated the role of ESE in asymptomatic AS, identifying a wide range of potential prognostic markers [12]. These include exercise-induced increases in transvalvular pressure gradients, elevation of left ventricular filling pressures (e.g., E/e’), the development of pulmonary hypertension, reduced contractile or diastolic reserve, impaired functional capacity, and alterations in myocardial strain. Despite these promising findings, results across studies have been heterogeneous, reflecting differences in study design, patient selection, stress protocols, and endpoint definitions. Consequently, the relative importance and reproducibility of these parameters remain uncertain, and their integration into clinical decision-making is not yet standardized.

Furthermore, contemporary management of AS is evolving rapidly, with increasing consideration of earlier intervention strategies, including transcatheter approaches [13]. In this changing landscape, refining risk stratification in asymptomatic patients has become even more relevant, as it may help identify individuals who could benefit from timely intervention before the onset of overt symptoms or irreversible cardiac damage.

Given these considerations, a comprehensive and structured synthesis of the available evidence is warranted. The aim of the present systematic review is to evaluate the prognostic value of ESE in patients with asymptomatic moderate and severe AS, with particular focus on stress-derived hemodynamic and functional parameters associated with adverse clinical outcomes. Additionally, this review seeks to provide an integrated overview of study methodologies, patient characteristics, and exercise-induced physiological responses in order to better define the role of ESE in contemporary risk stratification and management of asymptomatic AS.

## 2. Materials and Methods

This systematic review was conducted in accordance with the Preferred Reporting Items for Systematic Reviews and Meta-Analyses (PRISMA) guidelines [14] (Supplementary Materials File S1). The review protocol was prospectively registered on 28 March 2026 in the International Platform of Registered Systematic Review and Meta-analysis Protocols (INPLASY; registration number INPLASY202630105; Supplementary Materials File S2).

### 2.1. Search Strategy

A comprehensive literature search was independently performed by two investigators to identify studies evaluating the prognostic value of ESE in asymptomatic moderate and severe AS. Electronic databases, including PubMed, Scopus, and EMBASE, were systematically searched from database inception to March 2026.

The search strategy combined controlled vocabulary terms and free-text keywords related to AS and stress echocardiography. The following terms and their combinations were used: “aortic stenosis”, “asymptomatic”, “exercise stress echocardiography”, “stress echo”, “exercise testing”, “hemodynamics”, “prognosis”, “outcome”, and “risk stratification”. No restrictions were applied regarding language, publication date, or geographic location.

In addition, the reference lists of all eligible studies and relevant review articles were manually screened to identify further potentially relevant publications not captured through the electronic search. Discrepancies between reviewers during the screening process were resolved through discussion and consensus, with involvement of a third reviewer when necessary.

### 2.2. Eligibility Criteria

Studies were considered eligible if they met the following criteria: (i) an observational design (prospective or retrospective cohort studies); (ii) the inclusion of adult patients with asymptomatic moderate or severe AS; (iii) assessment performed using ESE based on physical exercise; and (iv) the reporting of clinical outcomes or prognostic endpoints, such as mortality, AVR, or composite cardiovascular events.

While the primary focus of this review was on asymptomatic moderate and severe AS, studies including closely related or overlapping populations (e.g., mild-to-moderate AS or paradoxical low-flow, low-gradient AS with preserved ejection fraction) were not systematically excluded when they provided relevant data on stress-induced hemodynamic responses and prognostic outcomes. These populations were considered clinically pertinent to the spectrum of asymptomatic AS and were interpreted within their specific pathophysiological context in the qualitative synthesis.

To ensure methodological consistency, only studies using exercise-based stress protocols (e.g., semi-supine bicycle or treadmill exercise) were included. Studies employing exclusively pharmacological stress modalities (e.g., dobutamine stress echocardiography) without an exercise component were excluded. Studies in which ESE represented the primary modality but included a minority of patients undergoing pharmacological stress (e.g., dobutamine) were considered eligible, provided that the overall study design, data acquisition, and outcome analyses were predominantly based on exercise-derived parameters. In these cases, the inclusion was deemed acceptable as the pharmacological component did not represent the main methodological approach and did not materially influence the overall interpretation of stress-induced findings.

Studies were also excluded if they did not provide extractable prognostic data, did not report clinical outcomes, or focused solely on diagnostic accuracy without outcome assessment. Additional exclusion criteria included (i) studies involving mixed populations without separable asymptomatic cohorts; (ii) studies focusing on other valvular diseases or non-AS populations; (iii) case reports, editorials, conference abstracts, letters, and narrative reviews; and (iv) preclinical or animal studies.

### 2.3. Study Selection and Data Extraction

Two investigators independently screened all retrieved records based on title and abstract. Full-text evaluation was subsequently performed for studies deemed potentially eligible, according to predefined inclusion and exclusion criteria. Any disagreement regarding study eligibility was resolved through consensus, with arbitration by a third reviewer when required.

Data extraction was performed independently using a standardized data collection form developed a priori. Extracted data included study characteristics (first author, year of publication, country, study design, and sample size), as well as methodological features related to stress protocols and workload increments.

Patient-level characteristics were also collected when available, including demographic variables (age, sex distribution), anthropometric parameters (body mass index, body surface area), cardiovascular risk factors (hypertension, diabetes, smoking status, dyslipidemia) and comorbidities.

Echocardiographic and hemodynamic parameters at rest and during exercise were systematically extracted. These included heart rate, blood pressure, transvalvular gradients, aortic valve area, left ventricular systolic and diastolic function indices, pulmonary pressures, myocardial deformation parameters, and functional capacity measures when available.

Clinical outcomes and follow-up data were also recorded, including event definitions, follow-up duration, and the main prognostic predictors identified in each study.

Given the heterogeneity in outcome definitions across studies, endpoints were extracted as originally reported, including mortality, AVR, and composite cardiovascular events. Because AVR represents a clinician-driven intervention rather than a spontaneous adverse event, endpoints including AVR were interpreted with caution, considering the potential influence of referral patterns and treatment decisions on event rates.

All extracted data were cross-checked for accuracy by both reviewers, and discrepancies were resolved through re-evaluation of the original articles.

### 2.4. Methodological Quality Assessment and Risk of Bias

The methodological quality and risk of bias of the included studies were independently assessed by two reviewers using the National Institutes of Health (NIH) Quality Assessment Tool for Observational Cohort and Cross-Sectional Studies [15].

This tool evaluates multiple methodological domains, including the clarity of study objectives, the definition of study population, exposure and outcome assessment, the consistency of measurement methods, and the adequacy of statistical analysis.

Each study was assessed across 14 predefined domains and rated as “Yes”, “No”, or “Not Reported”, according to NIH criteria. Overall study quality was classified based on the number of criteria fulfilled and the overall methodological rigor, with studies categorized as good, fair, or poor quality.

Disagreements in quality assessment were resolved through discussion and consensus. Results of the methodological appraisal were summarized using graphical representations of risk of bias.

### 2.5. Data Synthesis and Statistical Approach

Given the heterogeneity in study design, patient populations, stress protocols, and outcome definitions, a quantitative meta-analysis was not performed. Instead, a structured descriptive and qualitative synthesis of the available evidence was conducted.

To provide an overall characterization of the study population and physiological responses, pooled descriptive estimates were derived from study-level data. Continuous variables were summarized as weighted medians and corresponding interquartile ranges (IQRs), with weighting based on the sample size of each study. Since most studies reported continuous variables as mean ± standard deviation, underlying distributions were approximated assuming normality. These approximated distributions were subsequently used to derive pooled medians and dispersion measures, allowing for harmonization of heterogeneous reporting formats across studies.

This approach should be interpreted as an approximate descriptive synthesis rather than a formal statistical pooling method. The assumption of normality and reconstruction of distributions from summary statistics may introduce imprecision, particularly in the presence of skewed data or between-study heterogeneity. Accordingly, these pooled estimates are intended to provide a general overview of central tendency and variability across studies, without implying statistical inference or comparability equivalent to meta-analytic techniques.

Using this framework, summary estimates were generated for demographic, clinical, and echocardiographic parameters at rest and during exercise. Estimates were calculated only when sufficient data were available, and both the number of contributing studies and the corresponding pooled sample size were systematically reported to enhance transparency.

When both resting and peak exercise values were available, the relative change (Δ) between conditions was calculated and summarized using the same weighted approach. This allowed for a consistent evaluation of stress-induced physiological responses and provided insight into dynamic functional reserve across the included populations.

Given the observational nature of the included studies, clinical outcomes and prognostic predictors were synthesized qualitatively rather than quantitatively. Predictors identified across studies were interpreted within their respective pathophysiological context, including valvular hemodynamics, ventricular systolic and diastolic function, pulmonary circulation, myocardial deformation, and functional capacity. The frequency with which specific predictors were reported across studies was also assessed to identify the most consistent and reproducible markers of risk.

No formal statistical pooling of effect sizes, assessment of between-study heterogeneity, or evaluation of publication bias was performed, in line with the qualitative design of the review. Nevertheless, consistency of findings across studies, directionality of associations, and reproducibility of key predictors were carefully evaluated to strengthen the robustness of the overall interpretation.

All analyses were conducted at the study level, and no individual patient data were used. Data processing, aggregation, and descriptive analyses were performed using standard spreadsheet software (Microsoft Excel, Microsoft Corporation, Redmond, WA, USA), ensuring traceability and reproducibility of the analytical workflow.

### 2.6. Use of Artificial Intelligence for Language Editing

Artificial intelligence tools were used solely to support language editing during manuscript preparation. In particular, ChatGPT version GPT-5.3 (OpenAI, San Francisco, CA, USA) was utilized to improve grammar, spelling, and overall readability of the text.

No AI-based tools were employed for study design, literature search, study selection, data extraction, statistical analysis, or interpretation of results. All scientific content, analyses, and conclusions were independently developed and verified by the authors, who take full responsibility for the integrity and accuracy of the work.

## 3. Results

### 3.1. Study Selection

A total of 390 records were initially identified through PubMed, Scopus, and EMBASE databases. After removal of duplicates (n = 35), 355 records were screened based on title and abstract. Of these, 335 studies were excluded according to predefined eligibility criteria. The remaining 20 articles underwent full-text assessment. Nine studies were further excluded due to the absence of extractable prognostic data. Ultimately, 11 studies [16,17,18,19,20,21,22,23,24,25,26] were included in the systematic review, forming the final study population for qualitative synthesis.

**Figure 1:**
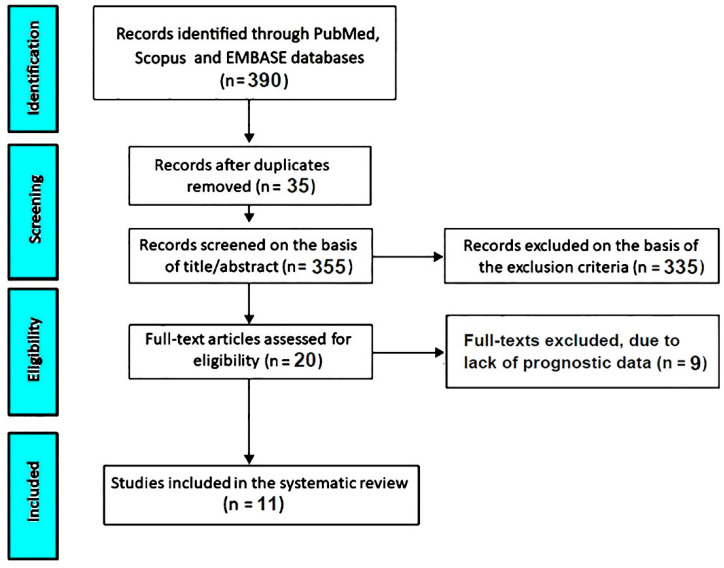
PRISMA flowchart of study selection.

The study selection process is summarized in Figure 1.

### 3.2. Study Characteristics

The main characteristics of the included studies are summarized in Table 1.

The 11 studies were published between 2005 and 2025 and were predominantly prospective in design, with most investigations conducted in a monocentric setting, although several multicenter cohorts were also included. Overall, the methodological approaches were relatively homogeneous, particularly with regard to patient selection and stress echocardiography protocols.

Exercise stress echocardiography was most commonly performed using a semi-supine bicycle protocol with stepwise incremental workloads, typically ranging from 10 to 25 W increases at regular intervals. Treadmill-based protocols were adopted in selected studies, while one study also incorporated dobutamine stress echocardiography. Across studies, echocardiographic acquisitions were mainly performed using commercially available systems from major vendors, predominantly GE and Philips platforms, with occasional use of Siemens systems.

The study populations mainly consisted of patients with asymptomatic moderate-to-severe or severe AS and preserved left ventricular ejection fraction. Sample sizes varied substantially across studies, ranging from small single-center cohorts to larger observational series. Despite this variability, patient selection criteria were broadly consistent, focusing on clinically stable, asymptomatic individuals undergoing risk stratification.

The prevalence of bicuspid aortic valve, when reported, was relatively low and heterogeneous across studies, with a median value of approximately 20% (IQR 13–27%), although several studies did not provide this information.

Overall, the included cohorts were predominantly male and encompassed a clinically representative spectrum of asymptomatic AS, including specific phenotypes such as paradoxical low-flow, low-gradient AS. This heterogeneity enhances the external validity of the findings while preserving a coherent clinical framework for evaluating ESE-derived prognostic markers.

### 3.3. Baseline Clinical Characteristics

Baseline demographic and clinical features are reported in Table 2.

Across the pooled population (n = 1647), patients were generally elderly (weighted median age 68.8 years) with a clear male predominance (64%). Cardiovascular risk factors were highly prevalent, particularly hypertension and dyslipidemia, while diabetes and prior cardiovascular events were less frequent but consistently represented across studies. Smoking history was also common, further reflecting a population with a substantial atherosclerotic burden.

Anthropometric parameters, when available, indicated a moderately elevated cardiometabolic profile, with median body mass index values in the overweight range and relatively consistent body surface area across cohorts. However, data completeness for these variables varied among studies, as did reporting of certain comorbidities such as chronic kidney disease.

Similarly, background medical therapy reflected contemporary management of cardiovascular risk, with frequent use of antiplatelets, statins, and renin–angiotensin system inhibitors. Beta-blockers were also commonly prescribed, whereas diuretics were less consistently reported, likely reflecting differences in baseline clinical status and reporting practices.

Overall, the included population represents a typical asymptomatic AS cohort with a substantial burden of cardiovascular comorbidities, providing a clinically relevant and externally valid framework for the evaluation of prognostic markers derived from ESE.

### 3.4. Rest and Exercise Echocardiographic Findings

Table 3 summarizes echocardiographic and hemodynamic parameters at rest and during stress.

At baseline, patients exhibited preserved left ventricular systolic function, with echocardiographic features consistent with moderate-to-severe AS. Overall, resting measurements confirmed a hemodynamically stable condition, with balanced ventricular volumes, preserved contractility, and no evidence of overt decompensation, supporting the classification of these individuals as asymptomatic despite significant valvular obstruction.

During exercise, a marked physiological response was observed, characterized by a coordinated increase in chronotropic and pressor responses, reflecting appropriate cardiovascular adaptation to increased metabolic demand. This was accompanied by a substantial rise in transvalvular gradients, consistent with the flow-dependent nature of AS severity and highlighting the dynamic interplay between valve obstruction and cardiac output during stress.

Importantly, several functional and hemodynamic parameters demonstrated clinically meaningful changes under stress conditions. In particular, indices of left ventricular filling pressure increased, suggesting a limited diastolic reserve and early elevation of intracardiac pressures during exercise. Similarly, pulmonary pressures rose significantly, indicating transmission of left-sided hemodynamic burden to the pulmonary circulation. These findings underscore the presence of subclinical functional impairment that may not be evident under resting conditions.

In contrast, aortic valve area showed minimal variation between rest and stress, reflecting the fixed anatomical component of valvular obstruction. This dissociation between stable anatomical severity and dynamic hemodynamic response reinforces the importance of functional assessment in AS.

Overall, ESE provided incremental functional and hemodynamic information beyond resting evaluation, allowing for a more comprehensive characterization of disease severity.

### 3.5. Clinical Outcomes and Prognostic Predictors

Clinical outcomes and their main predictors are detailed in Table 4.

Across studies, follow-up duration ranged from 12 to 83 months, with event rates consistently between 23% and 38%, reflecting a clinically relevant incidence of adverse outcomes despite the asymptomatic status of the populations. Although endpoint definitions varied across studies, the overall event burden remained comparable, supporting the robustness of prognostic analyses across different cohorts.

The most frequently reported endpoints included composite cardiovascular events, AVR, and mortality. A broad spectrum of prognostic markers emerged from ESE. Among these, exercise-induced increase in mean transaortic gradient (ΔMTPG) and exercise E/e’ratio were the most consistently identified predictors, reflecting the combined impact of valvular hemodynamics and left ventricular filling pressures under stress conditions. Additional relevant parameters included systolic pulmonary artery pressure during exercise, projected aortic valve area, functional capacity indices, and myocardial deformation parameters, highlighting the multidimensional nature of risk assessment in this setting.

**Figure 2:**
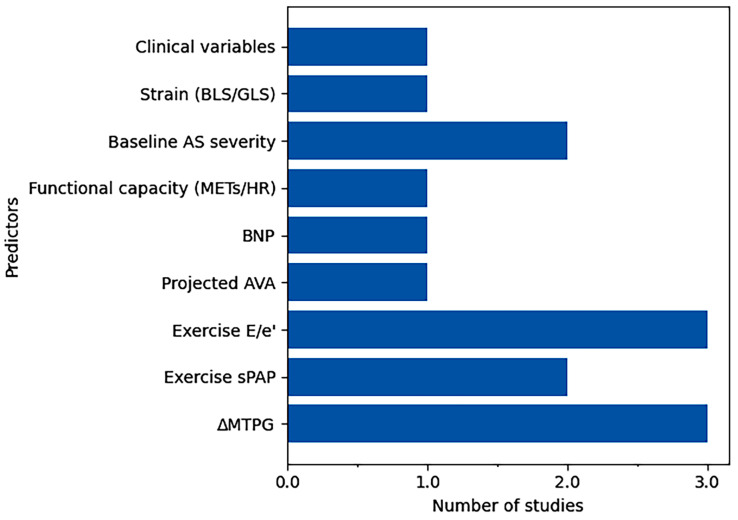
A bar chart illustrating the frequency of the main prognostic predictors reported across the included studies. AS, aortic stenosis; AVA, aortic valve area; BLS, basal longitudinal strain; BNP, B-type natriuretic peptide; E/e’, ratio of early mitral inflow velocity (E) to early diastolic mitral annular velocity (e’); GLS, global longitudinal strain; HR, heart rate; METs, metabolic equivalents; sPAP, systolic pulmonary artery pressure; ΔMTPG, change in mean transaortic pressure gradient.

As shown in Figure 2, prognostic markers were heterogeneously distributed across studies, with hemodynamic stress-derived parameters—particularly ΔMTPG and exercise E/e’—representing the most frequently reported and reproducible predictors. In contrast, clinical variables and baseline disease severity were less consistently identified, suggesting that dynamic functional assessment may provide incremental prognostic value beyond resting evaluation.

**Figure 3:**
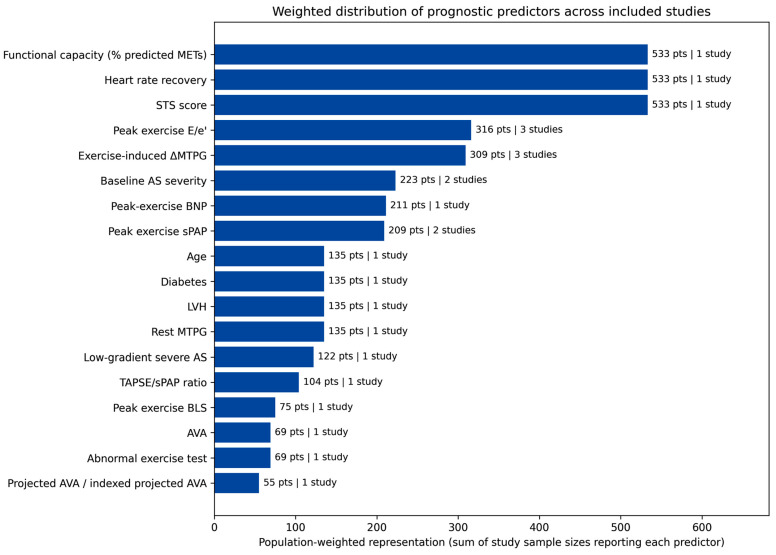
Population-weighted distribution of prognostic predictors across included studies. Each predictor was weighted according to the sample size of the study in which it was reported. Bar length represents the cumulative number of patients from studies identifying that predictor, providing a population-level estimate of its relative contribution. Labels indicate both the weighted sample size (number of patients) and the number of contributing studies. AS, aortic stenosis; AVA, aortic valve area; BLS, basal longitudinal strain; BNP, B-type natriuretic peptide; E/e’, ratio of early transmitral flow velocity to early diastolic mitral annular velocity; LVH, left ventricular hypertrophy; METs, metabolic equivalents; MTPG, mean transaortic pressure gradient; sPAP, systolic pulmonary artery pressure; STS, Society of Thoracic Surgeons; TAPSE, tricuspid annular plane systolic excursion.

However, this representation does not account for differences in sample size across studies. To address this limitation, a sample size-weighted analysis was performed (Figure 3), in which each predictor was weighted according to the number of patients in the study reporting it.

In the weighted analysis, predictors derived from larger cohorts—particularly functional capacity indices and global risk scores—showed a greater contribution to the overall distribution.

Notably, exercise-derived hemodynamic parameters such as ΔMTPG and E/e’ remained among the most relevant predictors even after weighting, confirming their robustness across both study-level frequency and population-weighted analyses.

Overall, these findings indicate that ESE-derived variables capture multiple pathophysiological domains, including valvular obstruction, ventricular diastolic function, contractile reserve, and cardiopulmonary interaction. The integration of these parameters within a stress-based evaluation framework allows for a more comprehensive and clinically meaningful risk stratification in moderate and severe asymptomatic AS, potentially improving identification of patients at higher risk of adverse outcomes despite preserved resting status.

### 3.6. Methodological Quality Assessment

**Figure 4:**
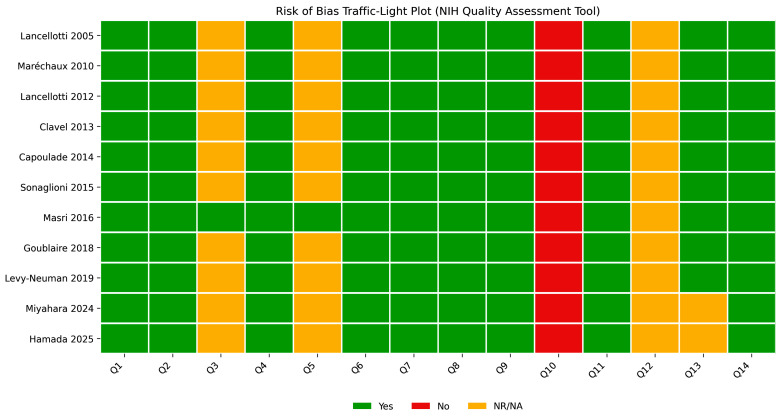
Traffic light plot of risk of bias. Visual representation of domain-specific quality assessment for each included study [16,17,18,19,20,21,22,23,24,25,26].

**Figure 5:**
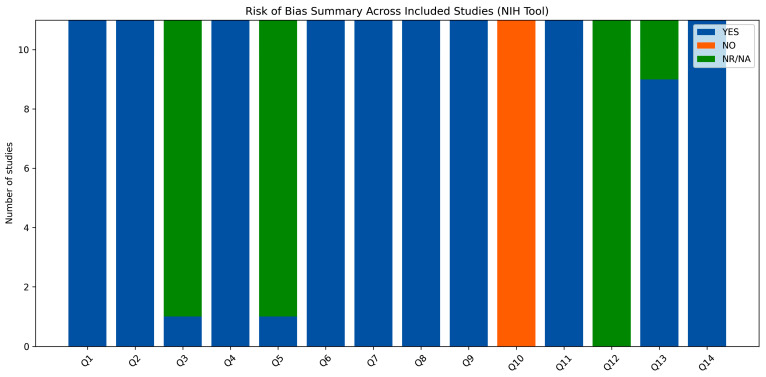
Risk of bias summary across studies [16,17,18,19,20,21,22,23,24,25,26]. Bar chart showing distribution of “yes”, “no”, and “not reported” ratings across methodological domains.

The methodological quality of the included studies is summarized in Table 5, with a graphical representation provided in Figure 4 and Figure 5.

Overall, all studies were rated as good quality according to the NIH assessment tool. The majority of domains showed a low risk of bias, particularly regarding study objectives, population definition, and exposure assessment. However, some methodological limitations were consistently observed, including incomplete reporting of sample size justification and limited information on blinding procedures. These aspects were reflected in the graphical summaries, where a small proportion of domains were classified as “not reported” or “no”. Despite these limitations, the overall methodological consistency across studies supports the robustness of the available evidence base.

## 4. Discussion

### 4.1. Main Findings

This systematic review provides a comprehensive synthesis of the available evidence on the prognostic role of ESE in patients with moderate and severe asymptomatic AS, highlighting the intrinsic limitations of a purely symptom-based clinical approach.

Across the included studies, a substantial proportion of patients classified as asymptomatic experienced adverse events during follow-up, reinforcing the concept that clinical silence does not equate to hemodynamic stability. This observation is consistent with earlier reports showing that AS may progress insidiously, with symptoms often under-recognized or delayed, particularly in elderly or sedentary individuals.

Within this context, ESE emerges as a powerful tool capable of revealing latent abnormalities that are not apparent at rest. The most consistent finding across studies is the prognostic relevance of dynamic parameters, particularly exercise-induced increases in transvalvular gradients and markers of elevated filling pressures. These findings extend early observations demonstrating that an abnormal rise in mean gradient during exercise reflects a more advanced disease state and is independently associated with adverse outcomes.

Importantly, the prognostic information provided by ESE appears to be inherently multidimensional, integrating valvular severity, myocardial function, and systemic adaptation to stress. This integrated response offers a more realistic representation of disease burden, explaining why stress-derived parameters often outperform resting measurements in predicting clinical evolution.

### 4.2. Pathophysiological Mechanisms of ESE Findings in as Patients

The pathophysiological basis underlying the prognostic value of exercise-derived parameters in AS lies in the dynamic interplay between fixed valvular obstruction and the adaptive capacity of the cardiovascular system [27]. Under resting conditions, compensatory mechanisms—particularly left ventricular hypertrophy—allow for the maintenance of cardiac output and preservation of ejection fraction, often masking early myocardial dysfunction [28]. However, exercise imposes an additional hemodynamic burden that may exceed these compensatory reserves [29].

The increase in transvalvular gradients observed during exercise reflects the flow-dependent nature of AS. In patients with reduced valve compliance or more advanced disease, the rise in cardiac output leads to a disproportionate increase in gradient, unmasking the functional severity of the stenosis [30]. This phenomenon has been consistently associated with adverse outcomes and reflects limited valvular reserve [31].

At the myocardial level, exercise-induced elevation of E/e’ ratio represents an early marker of impaired diastolic reserve [32]. Progressive myocardial fibrosis and increased chamber stiffness, which are well-described consequences of chronic pressure overload, contribute to an abnormal rise in filling pressures during stress, even when resting values remain within normal limits [33]. This elevation in filling pressures is transmitted backward to the pulmonary circulation, explaining the frequent development of exercise-induced pulmonary hypertension, a condition that has been shown to carry independent prognostic significance [34].

More broadly, these findings align with the concept of progressive “cardiac damage” in AS, in which the pathological process extends beyond the valve to involve the left ventricle, left atrium, pulmonary vasculature, and eventually the right ventricle [35,36]. Emerging evidence suggests that abnormalities in myocardial deformation and exercise capacity reflect this broader disease spectrum and may represent earlier markers of decompensation than conventional indices [37,38].

### 4.3. Clinical Implications

The present findings have relevant implications for the clinical management of asymptomatic moderate and severe AS, a setting in which decision-making remains complex and frequently relies on indirect indicators of disease severity. While current guideline recommendations are primarily based on symptom status and resting echocardiographic parameters, the evidence synthesized in this review supports a more dynamic and comprehensive approach to risk stratification.

Exercise stress echocardiography enables integrated assessment of valvular hemodynamics, ventricular performance, and cardiopulmonary adaptation under physiological stress. This approach facilitates the identification of patients who, despite the absence of symptoms, exhibit abnormal responses associated with an increased risk of adverse outcomes. In particular, stress-induced increases in transvalvular gradient, elevated filling pressures, development of pulmonary hypertension, and impaired functional capacity consistently emerge as clinically meaningful markers of risk.

Specific exercise-derived thresholds—such as E/e’ ≥ 15, an increase in mean transvalvular pressure gradient of approximately 18–20 mmHg or greater, and systolic pulmonary artery pressure ≥ 60 mmHg—appear to identify patients with limited hemodynamic reserve and early ventricular decompensation. Although not yet incorporated into formal guideline recommendations, the consistency of these findings across studies suggests that they may represent useful indicators of disease progression in clinical practice.

However, caution is warranted when interpreting exercise-derived E/e’ values in patients with significant mitral annular calcification (MAC), a condition frequently encountered in the AS population. MAC may alter mitral annular motion and impair left ventricular and atrial compliance, potentially leading to an overestimation of filling pressures when using tissue Doppler-derived indices. Therefore, E/e’ thresholds should be interpreted within the broader clinical and echocardiographic context, particularly in the presence of advanced annular calcification [39].

Within this framework, the integration of ESE findings into clinical decision-making supports a more individualized follow-up strategy. Patients with a normal stress response may be safely managed with guideline-recommended surveillance intervals, whereas those with abnormal findings may benefit from closer monitoring, with shorter reassessment intervals aimed at capturing early clinical or structural deterioration. In this context, ESE provides incremental information beyond resting evaluation, allowing earlier recognition of patients transitioning toward a more advanced disease stage.

**Figure 6:**
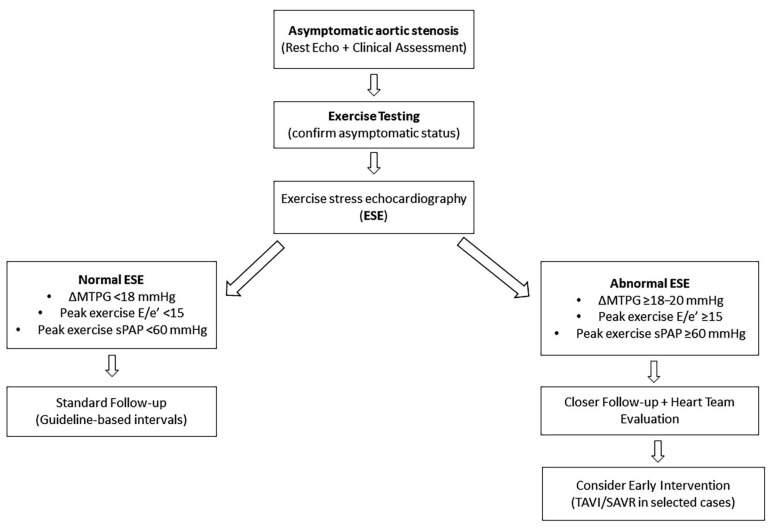
The proposed practical algorithm for the use of exercise stress echocardiography in asymptomatic aortic stenosis. A schematic representation of a stepwise clinical approach integrating exercise stress echocardiography (ESE) into the management of patients with asymptomatic aortic stenosis. Following initial clinical and resting echocardiographic evaluation, exercise testing is used to confirm asymptomatic status. ESE allows for the assessment of dynamic hemodynamic responses under physiological stress. Patients with normal ESE findings (ΔMTPG < 18 mmHg, peak exercise E/e’ < 15, and sPAP < 60 mmHg) may continue guideline-based follow-up. In contrast, the presence of abnormal stress-induced parameters (ΔMTPG ≥ 18–20 mmHg, E/e’ ≥ 15, or sPAP ≥ 60 mmHg) identifies a higher-risk subgroup that may benefit from closer surveillance and early multidisciplinary Heart Team evaluation, with consideration of earlier intervention in selected cases; ESE, exercise stress echocardiography; ΔMTPG, exercise-induced change in mean transvalvular pressure gradient; E/e’, ratio of early transmitral flow velocity to early diastolic mitral annular velocity; sPAP, systolic pulmonary artery pressure; TAVI, transcatheter aortic valve implantation; SAVR, surgical aortic valve replacement.

A stepwise clinical approach incorporating these elements is proposed in Figure 6.

Following initial clinical and resting echocardiographic assessment, exercise testing should be performed to confirm asymptomatic status, and, when feasible, complemented by stress echocardiography to characterize dynamic responses. A normal stress profile supports routine follow-up, while abnormal findings—particularly significant increases in gradient, elevated filling pressures, or exercise-induced pulmonary hypertension—should prompt intensified surveillance and consideration of early referral to a multidisciplinary Heart Team. This is particularly relevant in the current era of expanding transcatheter treatment options.

Overall, this strategy reflects a shift toward a more proactive and personalized management of AS, with the aim of identifying high-risk patients before the onset of symptoms and irreversible myocardial damage.

### 4.4. Sources of Heterogeneity

The interpretation of the present findings should take into account several sources of heterogeneity inherent to the included studies. Variability in study design represents an important factor, as both prospective and retrospective cohorts were included, often with different inclusion criteria and referral patterns. This may have influenced patient selection and the observed event rates.

Additionally, the populations studied were not entirely homogeneous, encompassing a spectrum of AS severity ranging from moderate to severe, as well as specific subgroups such as low-flow, low-gradient phenotypes. These entities are characterized by distinct hemodynamic profiles and clinical trajectories, which may influence both baseline characteristics and response to exercise.

Methodological differences in stress protocols further contribute to heterogeneity. The use of semi-supine bicycle versus treadmill exercise, as well as differences in workload progression and termination criteria, may affect the magnitude of hemodynamic responses and the detection of abnormalities.

Finally, variability in echocardiographic techniques and outcome definitions, along with differences in follow-up duration, may have influenced the identification and relative importance of prognostic predictors. Despite these limitations, the consistency of key findings across studies supports the overall robustness of the conclusions.

### 4.5. Future Directions

Future research should aim to further refine the role of ESE within the clinical management of asymptomatic AS by addressing several unmet needs.

First, large-scale prospective studies are required to validate the prognostic significance of stress-derived parameters and to establish standardized thresholds for clinical decision-making. In particular, the identification of reproducible cut-off values for variables such as exercise-induced transvalvular gradient increase, E/e’ ratio, and pulmonary pressures remains a critical step toward broader clinical implementation.

In addition, the integration of advanced imaging techniques, including myocardial deformation analysis and right ventricular–pulmonary arterial coupling, may provide further insight into early cardiac dysfunction and improve risk stratification. The combination of ESE with cardiopulmonary exercise testing and circulating biomarkers could also enable a more comprehensive and multiparametric assessment of disease severity and functional reserve [40].

Emerging technologies, including artificial intelligence and machine learning, hold promise for enhancing data integration and identifying complex prognostic patterns that may not be apparent through conventional analysis.

Ultimately, future studies should focus on incorporating ESE-derived parameters into structured clinical algorithms and randomized trials to determine whether earlier intervention guided by stress-induced abnormalities can improve patient outcomes.

### 4.6. Strengths and Limitations

This systematic review provides a comprehensive and structured overview of the prognostic role of ESE in asymptomatic moderate and severe AS, integrating data from multiple studies and offering a clinically relevant synthesis of available evidence. The use of a standardized methodological approach, including PRISMA-based selection and formal quality assessment, enhances the reliability of the findings and ensures methodological rigor.

At the same time, several limitations should be acknowledged. The analysis is based on aggregated study-level data, which limit the ability to perform adjusted analyses or explore interactions between variables. The absence of a formal meta-analysis precludes quantitative estimation of effect sizes and heterogeneity. Furthermore, variability in study design, patient populations, stress protocols, and measurement techniques may have influenced the results.

In addition, the overall evidence base is entirely observational, including both prospective and retrospective cohorts, and is characterized by heterogeneity in stress protocols, outcome definitions, and reporting methods. Incomplete reporting across several methodological domains and variables may further limit the comprehensiveness of quality assessment and contribute to data gaps, and the possibility of publication bias cannot be excluded.

Exercise stress echocardiography is also subject to intrinsic methodological limitations, including technical difficulties in image acquisition during physical exercise, reliance on flow-dependent parameters, and the potential underestimation of peak hemodynamic severity when image acquisition is delayed [41,42]. Moreover, the technique may be limited by suboptimal acoustic windows, operator dependency, and the relatively low reproducibility of certain Doppler-derived measurements [43].

These limitations highlight the need for larger, standardized prospective studies to further clarify the role of ESE in this setting.

## 5. Conclusions

Exercise stress echocardiography provides clinically meaningful incremental prognostic information in patients with asymptomatic moderate and severe AS by revealing subclinical abnormalities that are not detectable at rest. The dynamic assessment of transvalvular hemodynamics, diastolic function, pulmonary pressures, and functional capacity allows for a more comprehensive evaluation of disease severity and cardiovascular reserve.

The available evidence consistently indicates that stress-induced parameters, particularly those reflecting hemodynamic burden and impaired ventricular adaptation, are associated with adverse outcomes and may improve risk stratification beyond traditional resting measurements.

The incorporation of exercise-derived data into routine clinical assessment may therefore support a more individualized approach to patient management and contribute to more timely identification of candidates for intervention.

Further prospective studies are warranted to standardize methodologies and define clinically actionable thresholds for risk stratification.

## Figures and Tables

**Table 1:** Characteristics of included studies [16,17,18,19,20,21,22,23,24,25,26] evaluating ESE in asymptomatic aortic stenosis.

Study	Study Design	Stress Protocol	Workload Protocol	AS Population	Size (% Males)
Lancellotti P. (2005), Belgium [16]	Prospective, monocentric	Semi-supine bicycle exercise	25 W initial, +25 W every 2 min	Asymptomatic severe AS, normal LVEF	69 (70%)
Maréchaux S. (2010), France [17]	Prospective, multicentric	Semi-supine bicycle exercise	20–25 W start, +20–25 W every 3 min	Asymptomatic moderate–severe AS, normal LVEF	135 (64%)
Lancellotti P. (2012), Belgium [18]	Prospective, monocentric	Semi-supine bicycle exercise	25 W initial, +25 W every 2 min	Asymptomatic severe AS, normal EF	105 (59%)
Clavel M. (2013), Canada/France/Belgium [19]	Prospective, multicentric	Semi-supine bicycle (67%) or dobutamine stress echo (33%)	Exercise: 20–25 W start, +20–25 W every 3 min; Dobutamine: 2.5 → 20 µg/kg/min	Paradoxical low-flow, low-gradient AS, preserved LVEF	55 (64%)
Capoulade R. (2014), Canada/Belgium [20]	Prospective, multicentric	Semi-supine bicycle exercise	Symptom-limited graded exercise	Asymptomatic moderate–severe AS, preserved LVEF	211 (64%)
Sonaglioni A. (2015), Italy [21]	Prospective, monocentric	Semi-supine bicycle exercise	25 W initial, +25 W every 2 min	Asymptomatic mild-to-moderate isolated AS	90 (61%)
Masri A. (2016), USA [22]	Retrospective, monocentric	Treadmill (Bruce, modified Bruce, Cornell, Naughton)	Symptom-limited graded exercise	Asymptomatic severe AS, preserved LVEF	533 (78%)
Goublaire C. (2018), France [23]	Prospective, monocentric	Semi-supine bicycle exercise	Symptom-limited, +20–30 W every 1–3 min	Asymptomatic moderate-to-severe AS, preserved LVEF	148 (74%)
Levy-Neuman S. (2019), Israel [24]	Prospective, monocentric	Treadmill (Bruce protocol)	Symptom-limited graded exercise	Asymptomatic moderate-to-severe AS, preserved LVEF	75 (63%)
Miyahara D. (2024), Japan [25]	Retrospective, monocentric	Semi-supine bicycle exercise	10 W start, +10 W every 3 min	Asymptomatic moderate + low-gradient severe AS, LVEF ≥ 50%	122 (48%)
Hamada A. (2025), Japan [26]	Prospective, monocentric	Semi-supine bicycle exercise + CPET	Incremental ramp protocol	Asymptomatic severe AS, preserved LVEF	104 (57%)

AS, aortic stenosis; CPET, cardiopulmonary exercise testing; EF, ejection fraction; ESE, exercise stress echocardiography; LVEF, left ventricular ejection fraction; min, minutes; µg/kg/min, micrograms per kilogram per minute; W, watts.

**Table 2:** Baseline demographic and clinical characteristics of the pooled study population.

Parameter	Weighted Median	Weighted IQR (Q1–Q3)	Studies Included	Size (n)
Age (years)	68.8	60.1–77.7	11	1647
BSA (m^2^)	1.7	1.6–1.9	6	732
BMI (kg/m^2^)	27.2	24.2–30.0	4	1027
% males	64.0	61.1–78.0	11	1647
Hypertension (%)	69.0	53.0–69.0	10	1499
Diabetes (%)	16.0	14.0–19.0	11	1647
Smoking (%)	49.0	27.0–49.0	7	1187
Dyslipidemia (%)	58.0	46.0–69.0	10	1499
Obesity (%)	34.0	34.0–34.0	2	160
CKD (%)	0.0	0.0–7.0	3	356
CAD (%)	31.0	22.0–44.0	5	982
Previous stroke (%)	5.0	5.0–5.0	1	533
Aspirin (%)	59.0	59.0–59.0	1	533
Beta-blockers (%)	41.0	38.8–41.0	3	745
ACE-i (%)	36.0	36.0–39.3	3	745
Diuretics (%)	8.2	8.2–28.8	2	212
Statins (%)	56.0	56.0–56.0	1	533

Data are presented as weighted medians with corresponding interquartile ranges (IQR; Q1–Q3), with weighting based on the sample size of each study. The column “Studies Included” refers to the number of studies contributing data for each variable, while “Size (n)” indicates the overall number of patients considered for that specific parameter. ACE-i, angiotensin-converting enzyme inhibitor; BMI, body mass index; BSA, body surface area; CAD, coronary artery disease; CKD, chronic kidney disease; IQR, interquartile range; Q1, first quartile; Q3, third quartile.

**Table 3:** Pooled echocardiographic and hemodynamic parameters at rest and during exercise stress in asymptomatic aortic stenosis.

Parameter	Rest	Peak Exercise	Δ (Exercise–Rest)	Studies (n)
HR (bpm)	68.5 (60.7–77.1)	125.2 (110.5–141.3)	60.3 (40.5–78.7)	7 (1279)
SBP (mmHg)	138.9 (125.1–150.5)	168.9 (149.8–187.2)	33.7 (13.5–52.4)	7 (1285)
DBP (mmHg)	77.9 (69.3–84.9)	83.0 (72.3–93.0)	9.2 (−3.1–19.3)	4 (495)
RWT	0.5 (0.4–0.6)	—	—	1 (211)
LVMi	108.3 (84.8–136.8)	—	—	6 (1180)
LVEDD (mm)	44.4 (40.2–47.9)	—	—	2 (197)
LVESD (mm)	24.4 (20.4–28.4)	—	—	3 (730)
LVEDV (ml)	87.3 (70.7–106.9)	86.5 (68.3–105.3)	−3.9 (−22.8–15.9)	3 (296)
LVESV (ml)	29.5 (20.8–39.4)	27.0 (19.2–35.5)	−3.5 (−13.7–8.1)	3 (296)
LVEF (%)	61.8 (57.5–67.6)	68.5 (62.1–75.4)	4.5 (−1.5–11.2)	11 (1647)
SV (ml)	71.1 (57.7–86.1)	82.5 (65.8–101.6)	7.3 (−6.4–22.8)	5 (1056)
LV-GLS (%)	16.2 (14.0–18.1)	17.3 (15.1–19.1)	1.9 (−1.5–5.3)	3 (284)
LV-BLS (%)	17.0 (14.4–20.1)	17.8 (15.3–20.6)	2.1 (−2.3–5.1)	1 (75)
LASr	21.9 (16.7–24.9)	—	—	1 (104)
RV-FWLS	22.0 (18.1–26.2)	—	—	1 (104)
LA area	21.2 (17.2–24.9)	20.0 (16.5–23.7)	−3.4 (−10.7–2.8)	4 (460)
LAVi	42.6 (27.5–50.6)	—	—	1 (104)
E/A	0.8 (0.7–1.0)	1.1 (0.9–1.4)	0.3 (0.1–0.6)	4 (392)
E/e’	12.2 (9.0–15.3)	16.6 (12.4–21.1)	3.3 (−2.4–8.2)	6 (707)
sPAP (mmHg)	33.3 (26.6–39.3)	50.3 (39.1–62.7)	19.7 (6.8–31.5)	8 (1246)
TAPSE (mm)	19.9 (16.8–22.2)	23.7 (19.6–27.6)	3.1 (0.0–8.1)	2 (226)
Peak transaortic PG (mmHg)	60.5 (44.0–77.9)	82.9 (65.4–104.1)	19.7 (6.0–35.3)	6 (699)
Mean transaortic PG (mmHg)	35.6 (26.7–45.4)	46.0 (34.8–57.3)	10.4 (0.0–20.3)	11 (1647)
Aortic valve area (cm^2^)	0.9 (0.7–1.1)	0.9 (0.7–1.1)	0.04 (−0.16–0.22)	11 (1647)
BNP (pg/mL)	59.7 (23.5–111.4)	67.1 (20.3–103.8)	15.5 (−40.4–57.9)	2 (315)
METs	—	4.9 (4.0–5.7)	—	1 (105)
%FCMT	—	63.3 (55.4–72.6)	—	1 (105)

Values are reported as weighted medians with corresponding interquartile ranges (IQR; Q1–Q3), with weighting based on the sample size of each study. Resting values, peak exercise measurements, and their relative changes (Δ: exercise minus rest) are presented when available. The column “Studies (n)” indicates the number of studies contributing data for each parameter, with the corresponding total sample size provided in parentheses. All parameters were derived from standard transthoracic echocardiographic and hemodynamic assessments performed at rest and during exercise stress testing in patients with asymptomatic aortic stenosis. Missing values reflect parameters not consistently reported during stress conditions. BLS, basal longitudinal strain; BNP, B-type natriuretic peptide; DBP, diastolic blood pressure; E/A, ratio of early (E) to late (A) transmitral flow velocity; E/e’, ratio of early transmitral flow velocity to early diastolic mitral annular velocity; GLS, global longitudinal strain; HR, heart rate; LA, left atrium; LASr, left atrial reservoir strain; LAVi, left atrial volume index; LVEDD, left ventricular end-diastolic diameter; LVEDV, left ventricular end-diastolic volume; LVEF, left ventricular ejection fraction; LVESD, left ventricular end-systolic diameter; LVESV, left ventricular end-systolic volume; LVMi, left ventricular mass index; METs, metabolic equivalents; PG, pressure gradient; RV-FWLS, right ventricular free-wall longitudinal strain; RWT, relative wall thickness; SBP, systolic blood pressure; sPAP, systolic pulmonary artery pressure; SV, stroke volume; TAPSE, tricuspid annular plane systolic excursion; %FCMT, percentage of forecasted maximal theoretical capacity.

**Table 4:** Clinical outcomes, follow-up duration, and main prognostic predictors across included studies [16,17,18,19,20,21,22,23,24,25,26].

Study Name	Size (n)	Follow-Up (Months)	Events (Rate %)	Endpoint	Main Predictors
Lancellotti P. [16]	69	24	25 (36%)	Cardiac events (AVR, death)	ΔMTPG ≥ 18 mmHg; abnormal test; AVA ≤ 0.75 cm^2^
Maréchaux S. [17]	135	20	37 (27%)	Cardiac events (AVR, death)	Age ≥ 65 yrs; diabetes; LVH; rest MTPG > 35 mmHg; ΔMTPG
Lancellotti P. [18]	105	24	38 (36%)	Cardiac events (AVR, death)	Peak exercise sPAP ≥ 60 mmHg; ΔMTPG
Clavel M. [19]	55	12	21 (38%)	Adverse events (AVR, death)	Projected AVA; indexed projected AVA
Capoulade R. [20]	211	18	69 (33%)	Death or AVR	Peak exercise BNP
Sonaglioni A. [21]	90	12	28 (31%)	Cardiovascular events	Peak exercise E/e’ ≥ 15
Masri A. [22]	533	83	123 (23%)	All-cause mortality	STS score; % predicted METs; HR recovery
Goublaire C. [23]	148	14	44 (30%)	Clinical events	Baseline AS severity
Levy-Neuman S. [24]	75	34	19 (25%)	Cardiovascular events	Baseline AS severity; peak exercise BLS < 18%
Miyahara D. [25]	122	36	41 (34%)	AS-related events	Peak exercise E/e’ ≥ 15
Hamada A. [26]	104	24	32 (31%)	Composite endpoint	Peak exercise E/e’; sPAP; TAPSE/sPAP ratio

This table summarizes study characteristics related to clinical outcomes, including sample size, duration of follow-up, event rates, endpoint definitions, and the main predictors identified in each study. Endpoints varied across studies and included composite cardiovascular events, aortic valve replacement (AVR), and mortality. Reported predictors derive from clinical, echocardiographic, and exercise stress echocardiography (ESE) parameters. AS, aortic stenosis; AVR, aortic valve replacement; AVA, aortic valve area; BLS, basal longitudinal strain; BNP, B-type natriuretic peptide; E/e’, ratio of early transmitral flow velocity to early diastolic mitral annular velocity; HR, heart rate; LVH, left ventricular hypertrophy; METs, metabolic equivalents; MTPG, mean transaortic pressure gradient; sPAP, systolic pulmonary artery pressure; STS, Society of Thoracic Surgeons score; TAPSE, tricuspid annular plane systolic excursion; ΔMTPG, exercise-induced change in mean transaortic pressure gradient.

**Table 5:** Risk of bias assessment using the NIH Quality Assessment Tool for the included studies [16,17,18,19,20,21,22,23,24,25,26].

Study Name	Q1	Q2	Q3	Q4	Q5	Q6	Q7	Q8	Q9	Q10	Q11	Q12	Q13	Q14	Overall
Lancellotti P. [16]	Y	Y	NR	Y	NR	Y	Y	Y	Y	N	Y	NR	Y	Y	Good
Maréchaux S. [17]	Y	Y	NR	Y	NR	Y	Y	Y	Y	N	Y	NR	Y	Y	Good
Lancellotti P. [18]	Y	Y	NR	Y	NR	Y	Y	Y	Y	N	Y	NR	Y	Y	Good
Clavel M. [19]	Y	Y	NR	Y	NR	Y	Y	Y	Y	N	Y	NR	Y	Y	Good
Capoulade R. [20]	Y	Y	NR	Y	NR	Y	Y	Y	Y	N	Y	NR	Y	Y	Good
Sonaglioni A. [21]	Y	Y	NR	Y	NR	Y	Y	Y	Y	N	Y	NR	Y	Y	Good
Masri A. [22]	Y	Y	Y	Y	Y	Y	Y	Y	Y	N	Y	NR	Y	Y	Good
Goublaire C. [23]	Y	Y	NR	Y	NR	Y	Y	Y	Y	N	Y	NR	Y	Y	Good
Levy-Neuman S. [24]	Y	Y	NR	Y	NR	Y	Y	Y	Y	N	Y	NR	Y	Y	Good
Miyahara D. [25]	Y	Y	NR	Y	NR	Y	Y	Y	Y	N	Y	NR	NR	Y	Good
Hamada A. [26]	Y	Y	NR	Y	NR	Y	Y	Y	Y	N	Y	NR	NR	Y	Good

Symbols represent the assessment of each methodological quality domain according to the NIH Quality Assessment Tool for Observational Cohort and Cross-Sectional Studies. Specifically, Y (Yes) indicates that the criterion was fulfilled, N (No) indicates that the criterion was not fulfilled, and NR (Not Reported) indicates that the information was not available or insufficiently described in the original study. Domains Q1–Q14 correspond to the individual items of the NIH quality assessment checklist. The Overall rating reflects the global methodological quality of each study, classified as Good, Fair, or Poor based on the number of criteria satisfied and overall methodological rigor.

## Data Availability

Data extracted from included studies will be publicly available on Zenodo (https://zenodo.org, accessed on 9 April 2026).

## Supplementary Materials

The following supporting information can be downloaded at: https://www.mdpi.com/article/10.3390/jcm15093247/s1, Supplementary Materials File S1: PRISMA 2020 checklist; Supplementary Materials File S2.
