# Supplementary material for: The Prognostic Value of Exercise Stress Echocardiography in Asymptomatic Moderate and Severe Aortic Stenosis: A Systematic Review of Stress-Derived Hemodynamic and Functional Markers
Source: J Clin Med. 2026 Apr 24;15(9):3247. doi: 10.3390/jcm15093247 (PMC13163296; doi:10.3390/jcm15093247)
Supplement: Supplementary file 1 [file jcm-15-03247-s001.zip › Supplementary Materials S2.pdf]

International Platform of Registered Systematic Review and Meta-analysis Protocols

INPLASY

INPLASY202630105
doi: 10.37766/inplasy2026.3.0105
Received: 28 March 2026
Published: 28 March 2026

Corresponding author:
Andrea Sonaglioni

sonaglioniandrea@gmail.com

Author Affiliation:
IRCCS MultiMedica.

Prognostic Role of Exercise Stress Echocardiography in Asymptomatic Moderate and Severe Aortic Stenosis: A Systematic Review of Stress-Derived Hemodynamic and Functional Markers

Sonaglioni, A; Lombardo, M; Gramaglia, GF; Nicolosi GL; Baravelli, M.

ADMINISTRATIVE INFORMATION

Support - Ministero della Salute Ricerca Corrente.

Review Stage at time of this submission - Completed but not published.

Conflicts of interest - None declared.

INPLASY registration number: INPLASY202630105

Amendments - This protocol was registered with the International Platform of Registered Systematic Review and Meta-Analysis Protocols (INPLASY) on 28 March 2026 and was last updated on 28 March 2026.

INTRODUCTION

Review question / Objective The aim of the present systematic review is to evaluate the prognostic value of exercise stress echocardiography (ESE) in patients with asymptomatic aortic stenosis (AS), with particular focus on stress-derived hemodynamic and functional parameters associated with adverse clinical outcomes. Additionally, this review seeks to provide an integrated overview of study methodologies, patient characteristics, and exercise-induced physiological responses, in order to better define the role of ESE in the contemporary risk stratification and management of asymptomatic AS.

Rationale Over the past two decades, several studies have investigated the role of ESE in asymptomatic AS, identifying a wide range of potential prognostic markers [12]. These include exercise-induced increases in transvalvular pressure gradients, elevation of left ventricular filling pressures (e.g., E/e′), development of pulmonary hypertension, reduced contractile or diastolic reserve, impaired functional capacity, and alterations in myocardial strain. Despite these promising findings, results across studies have been heterogeneous, reflecting differences in study design, patient selection, stress protocols, and endpoint definitions. Consequently, the relative importance and reproducibility of these parameters remain uncertain, and their integration into clinical decision-making is not yet standardized.
Furthermore, contemporary management of AS is evolving rapidly, with increasing consideration of earlier intervention strategies, including transcatheter approaches [13]. In this changing landscape, refining risk stratification in asymptomatic patients has become even more relevant, as it may help identify individuals who could benefit from timely intervention before the onset of overt symptoms or irreversible cardiac damage.
Given these considerations, a comprehensive and structured synthesis of the available evidence is warranted.

Sonaglioni et al. INPLASY protocol 202630105. doi:10.37766/inplasy2026.3.0105 Downloaded from https://inplasy.com/inplasy-2026-3-0105/

References
12. https://doi.org/10.3390/jcm13123495.
13. https://doi.org/10.4103/heartviews.heartviews_34_22.

Condition being studied Aortic stenosis (AS) represents the most prevalent valvular heart disease in developed countries and its incidence is steadily increasing in parallel with population aging [1]. While the clinical management of symptomatic severe AS is well established, with aortic valve replacement (AVR) providing clear survival benefit, the optimal management of asymptomatic patients remains a matter of ongoing debate [2].
The natural history of asymptomatic AS is highly variable and often unpredictable. Although the annual risk of sudden cardiac death in truly asymptomatic individuals is relatively low, a substantial proportion of patients may experience rapid disease progression, symptom onset, or irreversible myocardial damage before clinical recognition [3]. Importantly, symptom assessment in this population is inherently challenging. Many patients, particularly older adults, may unknowingly limit their physical activity, leading to underreporting of symptoms and delayed referral for intervention [4]. As a result, reliance on symptom status alone may be insufficient to guide optimal timing of AVR [5].
In this context, there has been increasing interest in identifying objective markers capable of detecting early functional impairment and improving risk stratification in asymptomatic AS. Exercise testing is currently recommended by international guidelines to unmask latent symptoms and abnormal blood pressure responses [6,7]. However, conventional exercise testing provides limited insight into the underlying hemodynamic and myocardial mechanisms [8].
Exercise stress echocardiography (ESE) offers a more comprehensive approach by integrating functional, hemodynamic, and structural assessment during physiological stress [9]. This technique enables real-time evaluation of transvalvular gradients, left ventricular systolic and diastolic function, pulmonary pressures, myocardial deformation, and cardiopulmonary interaction [10]. Through this multiparametric assessment, ESE has the potential to uncover subclinical abnormalities not detectable at rest, thereby providing incremental prognostic information [11].

References
1. https://doi.org/10.1093/eurheartj/ehaf194.
2. https://doi.org/10.3390/jcm14217549.
3. https://doi.org/10.1001/jamacardio.2020.2497.
4. https://doi.org/10.1093/ageing/afad145.
5. https://doi.org/10.1016/j.hjc.2025.06.004.
6. https://doi.org/10.1016/j.jcmg.2013.08.011.
7. https://doi.org/10.3390/jcm11174983.
8. https://doi.org/10.1161/CIRCULATIONAHA.116.025457.
9. https://doi.org/10.31083/RCM47079.
10. https://doi.org/10.3390/jcm14207424.
11. https://doi.org/10.3390/diagnostics13152527.

METHODS

Search strategy A comprehensive literature search was independently performed by two investigators to identify studies evaluating the prognostic value of exercise stress echocardiography in asymptomatic aortic stenosis. Electronic databases, including PubMed, Scopus, and EMBASE, were systematically searched from database inception to February 2026.
The search strategy combined controlled vocabulary terms and free-text keywords related to aortic stenosis and stress echocardiography. The following terms and their combinations were used: “aortic stenosis”, “asymptomatic”, “exercise stress echocardiography”, “stress echo”, “exercise testing”, “hemodynamics”, “prognosis”, “outcome”, and “risk stratification”. No restrictions were applied regarding language, publication date, or geographic location.
In addition, the reference lists of all eligible studies and relevant review articles were manually screened to identify further potentially relevant publications not captured through the electronic search. Discrepancies between reviewers during the screening process were resolved through discussion and consensus, with involvement of a third reviewer when necessary.

Participant or population Patients affected by asymptomatic moderate or severe AS.

Intervention Studies were considered eligible if they met the following criteria: (i) observational design (prospective or retrospective cohort studies); (ii) inclusion of adult patients with asymptomatic moderate or severe aortic stenosis; (iii) assessment performed using exercise stress echocardiography based on physical exercise; and (iv) reporting of clinical outcomes or prognostic endpoints, such as mortality, aortic valve replacement, or composite cardiovascular events.
To ensure methodological consistency, only studies using exercise-based stress protocols (e.g., semi-supine bicycle or treadmill exercise) were included. Studies employing exclusively pharmacological stress modalities (e.g., dobutamine stress echocardiography) without an exercise component were excluded.

Sonaglioni et al. INPLASY protocol 202630105. doi:10.37766/inplasy2026.3.0105 Downloaded from https://inplasy.com/inplasy-2026-3-0105/

Comparator N/A.

Study designs to be included Observational Cohort and Cross-Sectional Studies.

Eligibility criteria Studies were considered eligible if they met the following criteria: (i) observational design (prospective or retrospective cohort studies); (ii) inclusion of adult patients with asymptomatic aortic stenosis; (iii) assessment performed using exercise stress echocardiography based on physical exercise; and (iv) reporting of clinical outcomes or prognostic endpoints, such as mortality, aortic valve replacement, or composite cardiovascular events.
To ensure methodological consistency, only studies using exercise-based stress protocols (e.g., semi-supine bicycle or treadmill exercise) were included. Studies employing exclusively pharmacological stress modalities (e.g., dobutamine stress echocardiography) without an exercise component were excluded.
Studies were also excluded if they did not provide extractable prognostic data, did not report clinical outcomes, or focused solely on diagnostic accuracy without outcome assessment. Additional exclusion criteria included: (i) studies involving mixed populations without separable asymptomatic cohorts; (ii) studies focusing on other valvular diseases or non-aortic stenosis populations; (iii) case reports, editorials, conference abstracts, letters, and narrative reviews; and (iv) preclinical or animal studies.

Information sources Electronic databases, including PubMed, Scopus, and EMBASE, were systematically searched from database inception to February 2026.

Main outcome(s) To evaluate the prognostic value of exercise stress echocardiography in patients with asymptomatic aortic stenosis, with particular focus on stress-derived hemodynamic and functional parameters associated with adverse clinical outcomes.

Additional outcome(s) Additionally, this review seeks to provide an integrated overview of study methodologies, patient characteristics, and exercise-induced physiological responses, in order to better define the role of ESE in the contemporary risk stratification and management of asymptomatic AS.

Data management Two investigators independently screened all retrieved records based on title and abstract. Full-text evaluation was subsequently performed for studies deemed potentially eligible, according to predefined inclusion and exclusion criteria. Any disagreement regarding study eligibility was resolved through consensus, with arbitration by a third reviewer when required.
Data extraction was performed independently using a standardized data collection form developed a priori. Extracted data included study characteristics (first author, year of publication, country, study design, and sample size), as well as methodological features related to stress protocols and workload increments.
Patient-level characteristics were also collected when available, including demographic variables (age, sex distribution), anthropometric parameters (body mass index, body surface area), and cardiovascular risk factors (hypertension, diabetes, smoking status, dyslipidemia, and comorbidities).
Echocardiographic and hemodynamic parameters at rest and during exercise were systematically extracted. These included heart rate, blood pressure, transvalvular gradients, aortic valve area, left ventricular systolic and diastolic function indices, pulmonary pressures, myocardial deformation parameters, and functional capacity measures when available.
Clinical outcomes and follow-up data were also recorded, including event definitions, follow-up duration, and the main prognostic predictors identified in each study.
When continuous variables were reported as mean ± standard deviation, values were recorded and subsequently used to derive summary distributions. For consistency of presentation across studies, continuous variables were expressed as weighted medians and interquartile ranges (IQRs), with weighting based on study sample size.
All extracted data were cross-checked for accuracy by both reviewers, and discrepancies were resolved through re-evaluation of the original articles.

Quality assessment / Risk of bias analysis The methodological quality and risk of bias of the included studies were independently assessed by two reviewers using the National Institutes of Health (NIH) Quality Assessment Tool for Observational Cohort and Cross-Sectional Studies. This tool evaluates multiple methodological domains, including clarity of study objectives, definition of study population, exposure and outcome assessment, consistency of measurement methods, and adequacy of statistical analysis.
Each study was assessed across 14 predefined domains and rated as "Yes", "No", or "Not Reported", according to NIH criteria. Overall study

Sonaglioni et al. INPLASY protocol 202630105. doi:10.37766/inplasy2026.3.0105 Downloaded from https://inplasy.com/inplasy-2026-3-0105/

quality was classified based on the number of criteria fulfilled and the overall methodological rigor, with studies categorized as good, fair, or poor quality.
Disagreements in quality assessment were resolved through discussion and consensus. Results of the methodological appraisal were summarized using graphical representations of risk of bias.

Strategy of data synthesis Given the heterogeneity in study design, patient populations, stress protocols, and outcome definitions, a quantitative meta-analysis was not performed. Instead, a structured descriptive and qualitative synthesis of the available evidence was conducted.
To provide an overall characterization of the study population and physiological responses, pooled descriptive estimates were derived from study-level data. Continuous variables were summarized as weighted medians and corresponding interquartile ranges (IQRs), with weighting based on the sample size of each study. Since most studies reported continuous variables as mean ± standard deviation, underlying distributions were approximated assuming normality. These approximated distributions were subsequently used to derive pooled medians and dispersion measures, allowing harmonization of heterogeneous reporting formats across studies.
This methodology enabled the generation of summary estimates for demographic, clinical, and echocardiographic parameters both at rest and during exercise, ensuring comparability across datasets while preserving the descriptive nature of the analysis . For variables reported in a limited number of studies, pooled estimates were calculated only when sufficient data were available, and the number of contributing studies alongside the corresponding pooled sample size was systematically reported to enhance transparency.
When both resting and peak exercise values were available, the relative change (Δ) between conditions was calculated and summarized using the same weighted approach. This allowed a consistent evaluation of stress-induced physiological responses and provided insight into dynamic functional reserve across the included populations.
Given the observational nature of the included studies, clinical outcomes and prognostic predictors were synthesized qualitatively rather than quantitatively. Predictors identified across studies were interpreted within their respective pathophysiological context, including valvular hemodynamics, ventricular systolic and diastolic function, pulmonary circulation, myocardial deformation, and functional capacity. The frequency with which specific predictors were reported across studies was also assessed to identify the most consistent and reproducible markers of risk.
No formal statistical pooling of effect sizes, assessment of between-study heterogeneity, or evaluation of publication bias was performed, in line with the qualitative design of the review. Nevertheless, consistency of findings across studies, directionality of associations, and reproducibility of key predictors were carefully evaluated to strengthen the robustness of the overall interpretation.
All analyses were conducted at the study level, and no individual patient data were used. Data processing, aggregation, and descriptive analyses were performed using standard spreadsheet software (Microsoft Excel, Microsoft Corporation, Redmond, WA, USA), ensuring traceability and reproducibility of the analytical workflow.

Subgroup analysis Not performed.

Sensitivity analysis Not performed.

Language restriction No.

Country(ies) involved Italy.

Keywords Exercise stress echocardiography; Risk stratification; Asymptomatic patients; Transvalvular gradient; Diastolic function; Pulmonary hypertension; Functional capacity; Prognosis.

Contributions of each author
Author 1 - Andrea Sonaglioni - Author 1 drafted the manuscript.
Email: sonaglioniandrea@gmail.com
Author 2 - Michele Lombardo - Supervision.
Email: michele.lombardo@multimedica.it
Author 3 - Giulio Francesco Gramaglia - The author 3 contributed to the search strategy.
Email: giulio.gramaglia@unimi.it
Author 4 - Gian Luigi Nicolosi - The author read, provided feedback and approved the final manuscript.
Email: gianluigi.nicolosi@gmail.com
Author 5 - Massimo Baravelli - Supervision.
Email: massimo.baravelli@multimedica.it

Sonaglioni et al. INPLASY protocol 202630105. doi:10.37766/inplasy2026.3.0105 Downloaded from https://inplasy.com/inplasy-2026-3-0105/
